# Fat Encapsulation Reduces Diarrhea in Piglets Partially by Repairing the Intestinal Barrier and Improving Fatty Acid Transport

**DOI:** 10.3390/ani11010028

**Published:** 2020-12-26

**Authors:** Min Tian, Jiaming Chen, Zhihui Wu, Hanqing Song, Fei Yang, Chang Cui, Fang Chen, Shihai Zhang, Wutai Guan

**Affiliations:** 1 Guangdong Provincial Key Laboratory of Animal Nutrition Control, College of Animal Science, South China Agricultural University, Guangzhou 510642, China; tianmin@stu.scau.edu.cn (M.T.); 201430690127@stu.scau.edu.cn (J.C.); wuzhihui@stu.scau.edu.cn (Z.W.); hqsong@stu.scau.edu.cn (H.S.); yfpg@163.com (F.Y.); cuichang1998a@163.com (C.C.); chenfang1111@scau.edu.cn (F.C.); 2 College of Animal Science and National Engineering Research Center for Breeding Swine Industry, South China Agricultural University, Guangzhou 510642, China; 3 Guangdong Laboratory for Lingnan Modern Agriculture, South China Agricultural University, Guangzhou 510642, China

**Keywords:** fat, intestinal barrier, fatty acid transport, piglets, AMPK signaling

## Abstract

**Simple Summary:**

Fat is an important energy resource in animal production. Studies of the effect of dietary fat on gut function will facilitate the development and utilization of new fat resources. In our previous study, we found that piglet diarrhea is related to the type of dietary fat ingested. Therefore, we wondered whether dietary fat regulates intestinal function by regulating the expression of key proteins in the piglet intestine. In this study, we confirmed that dietary fat regulates the expression of fatty acid transport, intestinal tight junctions, and β-defensin proteins. Moreover, we have shown for the first time that fat encapsulation reduces the incidence of diarrhea partially by alleviating the damage to intestinal mechanical and immunological barriers induced by fat with a low digestibility.

**Abstract:**

(1) Background: Nutritional strategies to enhance gut function and reduce the piglet diarrhea rate are critical to increase the growth performance of piglets. The purpose of this study was to investigate whether dietary fat types and/or fat microencapsulation techniques are involved in regulating the fatty acid transport system and the mechanical and immunological barriers of the small intestine. (2) Methods: Three hundred twenty-four weaning piglets were randomly divided into three groups fed a soybean oil diet (SBO, control group, 6.0% soybean oil), palm oil diet (PO, 6.0% palm oil), or encapsulated palm oil diet (EPO, 7.5% encapsulated palm oil). (3) Results: A significantly lower mRNA expression of the claudin was observed in the duodenum and jejunum of the PO group than in the SBO group (*p* < 0.05). However, the mRNA expression and protein abundance of claudin and ZO-1 in the jejunum of the EPO group were higher (*p* < 0.05) than in the PO group. Porcine β-defensin (pBD) secretion was not significantly different between the SBO and PO groups (*p* > 0.05), while the pBD-2 levels were significantly different (*p* < 0.05). Compared with the PO group, the EPO group exhibited a significantly increased secretion of pBD-2 and pBD-129 in the small intestine (*p* < 0.05) and pBD-1 in the jejunum and ileum (*p* < 0.05). The protein abundances of apolipoprotein AIV (Apo AIV) and intestinal fatty acid binding protein (I-FABP) were significantly lower in the PO group than in the SBO group (*p* < 0.05). Simultaneously, the protein abundances of fatty acid transport protein 4 (FATP4), fatty acid translocase (CD36), and I-FABP were higher in the EPO group than in the PO group. Furthermore, the low digestibility of palm oil (PO group) might negatively regulate intestinal tight junctions, fatty acid transporters, lipoproteins, and β-defensin through the activation of the AMPK/mTORC1 and AMPK/Sirt1/NF-κB pathways. (4) Conclusions: In summary, microencapsulation techniques might alleviate the negative effects of palm oil and help to improve the intestinal fatty acid transport system and barrier function.

## 1. Introduction

Dietary fat is a critical energy source for pigs. The use of an appropriate fat source is expected to increase the performance of pigs. Conversely, the dietary supplementation of fat with a low digestibility might jeopardize the growth of the pig due to an insufficient energy supply. Palm oil is a cheaper alternative oil source to soybean oil in animal husbandry. However, palm oil contains numerous saturated fatty acids and has a lower energy digestibility. Fat encapsulation may change the physical structure of oils and facilitate their digestion and absorption by piglets, which makes palm oil a potential feed ingredient for animals. 

The administration of fats with low digestible energy values might induce energy deficiency, retard the growth of pigs, and lead to intestinal dysfunction. The gut is the main site for the digestion and absorption of nutrients. In addition, the gut also forms the boundary between mammalian internal and external environments. The intestinal barrier is composed of a physical barrier, a chemical barrier, an immune barrier, and microorganisms, and these barriers jointly resist the invasion of foreign substances [1]. The maintenance of the integrity of intestinal barrier function is a key prerequisite to ensure the health of piglets [2,3,4]. As the predominant energy sensor in cells, adenosine 5′-monophosphate (AMP)-activated protein kinase (AMPK) is proposed to detect changes in cellular energy. Energy deprivation activates AMPK to regulate multiple biological functions upon the activation of numerous downstream signaling pathways. Nuclear factor kappa-B (NF-κB) is reported to be involved in the regulation of β-defensin expression [5,6]. Activation of AMPK leads to an increase in the sirtuin 1 (Sirt1) activity, which suppresses NF-κB-driven immune responses [7,8]. In addition, AMPK inhibits the activity of mechanistic target of rapamycin complex 1 (mTORC1) through the phosphorylation of the raptor on Ser792 [9]. Since mTORC1 is a master regulator of protein synthesis and cell proliferation [10], AMPK might regulate functional protein expression (tight junctions proteins, fatty acid transporters, etc.) in the intestine through the mTORC1 pathway. 

This study was designed to elucidate the effects of the dietary administration of soybean oil, palm oil, and encapsulated palm oil on intestinal function. As mentioned above, palm oil has a lower digestible energy which is expected to trigger the activation of AMPK and regulate gut function. In this study, palm oil impaired the expression of intestinal tight junction proteins, fatty acid transporters, and lipoproteins through the AMPK/mTORC1 pathway. At the same time, palm oil administration inhibited the β-defensin expression and activated AMPK/Sirt1/NF-κB signaling. Importantly, no significant difference in gut function was observed between soybean oil and encapsulated palm oil treatments, indicating that microencapsulation techniques could increase the digestibility of palm oil. These observations provide novel molecular biological evidence that encapsulated palm oil is an alternative energy source to soybean oil.

## 2. Materials and Methods

All the animal care and handling procedures conducted during this study were approved by the South China Agricultural University Animal Care and Use Committee (No. 20110107-1, Guangzhou, China). 

### 2.1. Diets

Three experimental diets based on corn and soybean meal are shown in Table S1: soybean oil diet (SBO, control group, 6.0% soybean oil), palm oil diet (PO, 6.0% palm oil), and encapsulated palm oil diet (EPO, 7.5% encapsulated palm oil). All the diets meet the nutrient requirements of 7 to 11 kg piglets [11]. Soybean oil was obtained from Cargill Grain and Oil Co., Ltd. (Dongguan, China). Palm oil was purchased from Yihai Kerry Grain & Oil Ltd. (Guangzhou, China). The same source of palm oil was encapsulated using 8.5% dried casein and 11.5% whey powder. The three ingredients were stirred at 60 °C for 30 min to uniformly mixed them in liquid form, pasteurized at 65 °C for 30 min, and homogenized at a pressure of 250 kg/cm^2^ before spray drying (EYELA Spray Drier SD-1000, Tokyo Rikakikai Co., Ltd., Tokyo, Japan). The inlet temperature of the drier was 180 °C and the outlet temperature was 80 °C. The size of the dried fat powder particles ranged from 30 to 50 μm. The fatty acid composition of the fat sources used in the experiment was analyzed using gas chromatography (GC-2010 Pro, SHIMADZU Corporation, Kyoto, Japan) and is shown in Table S2. 

### 2.2. Animals and Experimental Design

After a 7-day adaptation period, 324 weaning piglets (8.23 ± 0.22 kg, Duroc × Landrace × Large White) were randomly classified into the three treatment groups with six replicates (pens) per treatment and eighteen piglets per replicate based on their initial body weight and sex, and the experiment lasted for 28 days. All the piglets were provided with *ad libitum* access to feed and fresh water during the experiment. One heathy piglet of each replicate was sacrificed by electrocution on day 28. The piglets were selected for slaughter according to body weight (close to average weight) and sex (half of each). The duodenum, jejunum, and ileum were collected, snap-frozen in liquid nitrogen, and then stored at −80 °C until further analysis.

### 2.3. Data Collection

The initial body weight and final body weight were measured on days 0 and 28, respectively, with the pen as the replicate. During the trial, the number of the piglets with diarrhea symptoms was recorded by the attending veterinarian. Diarrhea symptoms were scored using the fecal scoring system (0—normal, hard form; 1—soft feces, soft form; 2—mild diarrhea, no form, loose, puddles; 3—severe diarrhea, watery) reported by Ren, et al. (2020) [12]. The diarrhea incidence is the ratio of the total number of diarrhea events to the product of the total piglet number and the number of test days. At the end of the experiment, the total feed intake of each group was measured.

### 2.4. Chemical Analyses

The gross energy (GE) of the feed sample was analyzed using an adiabatic oxygen bomb calorimeter (IKA C200, IKA Company, Staufen, Germany). The crude protein (CP) content was measured according to the method of the Association of Official Analytical Chemists [13]. The dietary ether extract was determined using the method described by Thiex, et al. (2003) [14]. Moreover, the dietary contents AAs was analyzed using ion-exchange chromatography (Hitachi L-8800 amino acid analyzer, Tokyo, Japan) [13]. The tissues (20–50 mg) of duodenum, jejunum, and ileum were rinsed with 1× PBS and homogenized in 1 mL of 1× lysis buffer. Then, the homogenate was broken with ultrasound until it was clear and centrifuged for 5 min at 10,000× *g* and 4 °C. The supernatant was obtained and assayed immediately. The concentrations of pBD-1, pBD-2, pBD-3, pBD-114, and pBD-129 were measured using commercially available ELISA kits from USCN Life Sciences (Wuhan, China), Shanghai Renjie Biotechnology (Shanghai, China) and Research Biological Technology (Shanghai, China), respectively. These kits are sandwich enzyme immunoassays for in vitro quantitative analysis. The absorbance of the colored product was determined using a Multiskan GO Microplate Spectrophotometer (Thermo Fisher Scientific, Rockford, IL, USA). The pBD concentrations of the samples were calculated with reference to the standard curve obtained from the standards of known concentrations.

### 2.5. RNA Extraction and Relative Qualification of mRNA

Total RNA was extracted from intestinal tissues using the RNAiso Plus reagent (Takara, Dalian, China). The concentration and purity of the RNA samples were assessed with a NanoDrop spectrophotometer (NanoDrop Technologies, Wilmington, DE, USA), and only RNA samples with an absorbance ratio (260/280) greater than 1.8 were used. Subsequently, RNA was reverse-transcribed into cDNA using the PrimeScript First Strand cDNA Synthesis Kit (Takara, Dalian, China) according to the manufacturer’s protocol. Real-time quantitative PCR (RT-qPCR) was performed using an ABI Prism 7500 sequence detection system (Applied Biosystems, Carlsbad, CA, USA) in a total 20 μL volume composed of 10 μL of RT-qPCR Master Mix (SYBR Green, Takara, Dalian, China), 2 μL of cDNAs, 0.8 μL of each PCR primer (10 μM), 0.4 of μL ROX (10 μ/μL, Takara), and 6 μL of dd water. The PCR program was set as follows: denaturation at 94 °C for 5 min followed by 40 repeated cycles of 94 °C for 30 s, 60 °C for 30 s, and 72 °C for 30 s. The relative gene expression was analyzed using the relative quantification (2^−ΔΔCt^) method. The primers used for RT-qPCR are shown in Table 1.

### 2.6. Western Blotting Analysis

Total protein was extracted from intestine tissues using the RIPA lysis buffer (Beyotime, Shanghai, China) supplemented with 1% of the protease inhibitor PMSF (100 mM) and 1% phosphatases inhibitor (100×). A BCA protein assay kit (Beyotime, Shanghai, China) was used to determine the total protein concentrations. Then, equal amounts of samples (20 μg of total protein) were resolved on 10% a polyacrylamide gel and transferred onto polyvinylidene difluoride (PVDF) membranes (Millipore, Bedford, MA, USA). After blocking with 5% skim milk for 2 h at room temperature, each membrane was washed five times with TBST buffer for 5 min each. Subsequently, the membranes were incubated at 4 °C overnight in TBST containing 5% BSA with the primary antibody. Antibodies against target proteins were obtained from commercial companies (Table 2). Subsequently, the membranes were incubated with the corresponding secondary antibodies (511203, Zen-Bio, Chengdu, China) at room temperature for 1.5 h. After five rinses, band signals were detected using a chemiluminescent ECL Western blot detection system (ProteinSimple, Santa Clara, CA, USA). Finally, the protein bands were quantified by densitometry using ImageJ software, and the relative protein abundance was normalized to total β-actin or total target protein levels (Image-Pro Plus 6.0) (Rockville, MD, USA).

### 2.7. Statistical Analysis

Data with values that exceeded 3 standard deviations from the mean were considered outliers and excluded from the analysis. All the data were tested for normality using the Shapiro–Wilk test. Statistical significance was analyzed with a one-way ANOVA using the MIXED procedure of the statistical software SAS version 9.4 (SAS Institute, Cary, NC, USA) with the pen as the experimental unit. Diet, sex, and their interaction were defined as the main effects. Means were generated and separated using the LSMEANS and PDIFF options. Statistical significance was considered if *p* ≤ 0.05 and trends were indicated at 0.05 < *p* ≤ 0.10. The results are presented as the means ± standard errors of the means.

## 3. Results

### 3.1. mRNA Expression and Protein Abundance of Tight Junction Proteins in the Intestine

**Figure 1:**
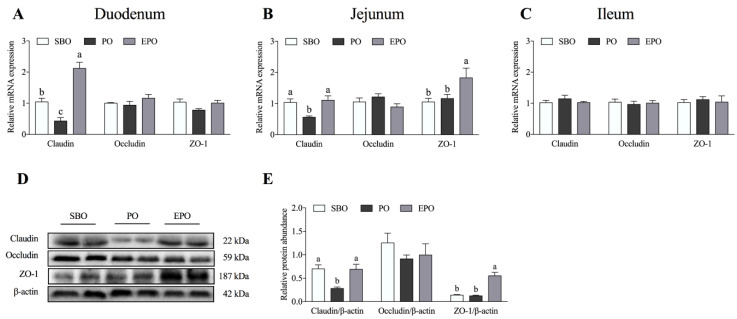
Expression and abundance of intestinal tight junction mRNAs and proteins. (**A**–**C**) The mRNA expression of tight junction proteins in the duodenum (**A**), jejunum (**B**), and ileum (**C**). (**D**,**E**) The abundance of tight junction proteins in the jejunum. SBO, soybean oil group; PO, palm oil group; EPO, encapsulated palm oil group. Different letters (a, b, and c) indicate a significant difference among different treatments (*p* < 0.05).

The mRNA expression of tight junction proteins (claudin, occludin, and ZO-1) in the duodenum, jejunum, and ileum is shown in Figure 1A–C. A significantly lower mRNA expression of claudin was detected in the duodenum of the PO group (*p* < 0.05) than in the SBO group. A significantly higher mRNA expression of claudin was observed in the duodenum of the EPO group (*p* < 0.05) than in the PO group. In the jejunum, the mRNA expression of claudin in the PO group was significantly lower (*p* < 0.05) than that in the SBO group. Simultaneously, the mRNA expression of claudin and ZO-1 was significantly (*p* < 0.05) higher than in the PO group. Furthermore, the mRNA expression of tight junction proteins in the ileum was not significantly different among the three groups in the ileum. 

Based on the mRNA results, we detected the protein abundance of tight junction proteins in the jejunum (Figure 1D,E). The protein abundance results were similar to those for the mRNA expression. A significantly lower protein abundance of claudin was detected (*p* < 0.05) in the PO group than in the SBO group. The protein abundances of claudin and ZO-1 were significantly higher in the EPO group (*p* < 0.05) than in the PO group. Additionally, the protein abundance of occludin was not significantly different among the three groups. 

### 3.2. Secretion of β-defensin in the Small Intestine of Piglets.

**Figure 2:**
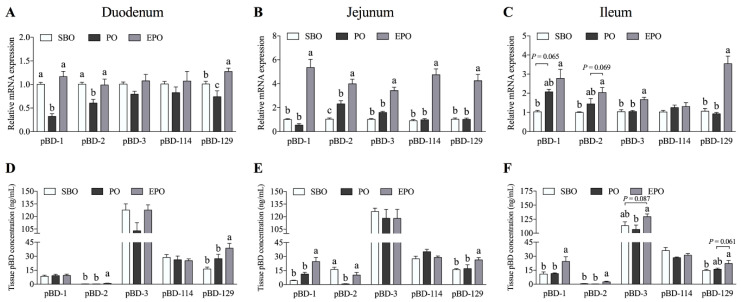
Secretion of β-defensins in the porcine small intestine. The mRNA expression and concentrations of porcine β-defensins in the duodenum (**A**,**D**), jejunum (**B**,**E**), and ileum (**C**,**F**). SBO, soybean oil group; PO, palm oil group; EPO, encapsulated palm oil group. Different letters (a, b, and c) indicate a significant difference among different groups (*p* < 0.05).

The differences in the intestinal porcine β-defensin expression (mRNA and tissue concentrations) among different treatments groups are shown in Figure 2. In the duodenum (Figure 2A,D), a lower mRNA expression of pBD-1, pBD-2, and pBD-129 was detected in the PO group than in the SBO group (*p* < 0.05). When animals were fed encapsulated palm oil, the mRNA expression of pBD-1 and pBD-2 was significantly higher in the EPO group than that in the PO group (*p* < 0.05) and showed no significant difference compared with the SBO group. Simultaneously, a significantly higher mRNA expression of pBD-129 was observed than in the other two groups (*p* < 0.05). Furthermore, the concentrations of pBD-2 and pBD-129 in the duodenal tissues were significantly higher than in the other two groups (*p* < 0.05). 

In the jejunum (Figure 2B,E), the mRNA expression of β-defensins in the EPO group was significantly higher than that in the other two groups (*p* < 0.05). Moreover, the concentrations of pBD-1 and pBD-129 in the jejunum were significantly higher than in the other two groups (*p* < 0.05). Interestingly, the defensin mRNA expression in the PO group and the EPO group was significantly higher than in the SBO group (*p* < 0.05). However, the pBD-2 concentration in the PO group was significantly lower than that in the other two groups (*p* < 0.05). No significant differences were observed between the SBO group and the EPO group. 

In the ileum (Figure 2C,F), the mRNA expression and secretion of five β-defensins were not significantly different between the SBO group and the PO group. However, the pBD-3 and pBD-129 mRNAs were expressed at significantly higher levels in the EPO group than in the other two groups. The expression of the pBD-2 mRNA in the EPO group showed a trend toward higher expression (*p* = 0.069) compared with the PO group. At the same time, the tissue concentrations of pBD-1, pBD-2, and pBD-3 in the EPO group were significantly higher (*p* < 0.05) than the PO group. The tissue concentration of pBD-129 in the EPO group showed a higher secretion trend (*p* = 0.061) than in the PO group.

### 3.3. Fatty Acid Transport System in the Jejunum Tissues

**Figure 3:**
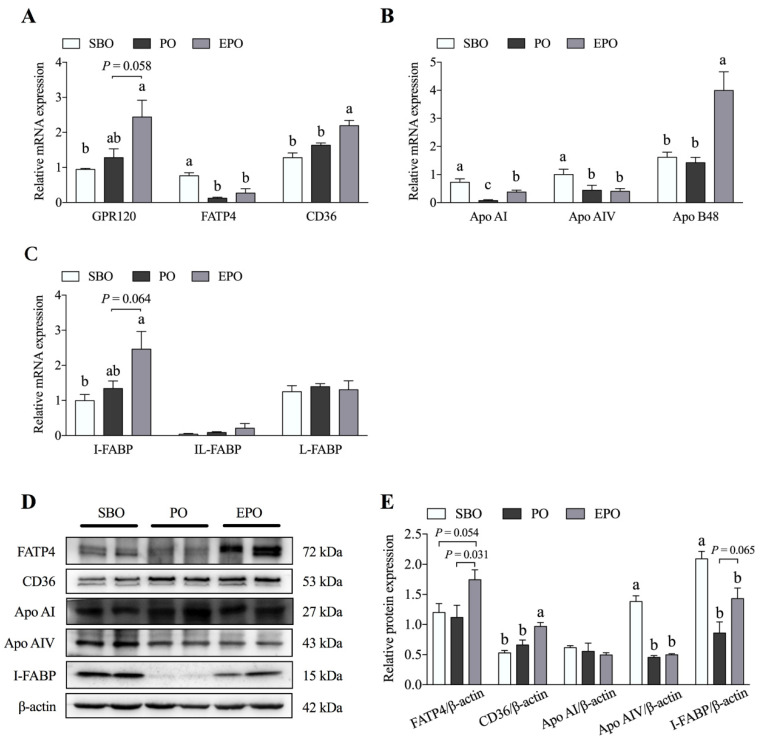
Expression of fatty acid transport system-related proteins in the porcine jejunum. The mRNA expression (**A**–**C**) and protein abundance (**D**,**E**) of proteins involved in fatty acid transport in the porcine jejunum. SBO, soybean oil group; PO, palm oil group; EPO, encapsulated palm oil group. Different letters (a, b, and c) indicate a significant difference among different groups (*p* < 0.05).

The mRNA expression and protein abundance of fatty acid transporters and lipoproteins are shown in Figure 3. Compared with the SBO group, the mRNA expression of fatty acid transport protein 4 (FATP4), apolipoprotein A-I (Apo AI), and apolipoprotein A-IV (Apo AIV) was significantly lower in the PO group (*p* < 0.05). The mRNA of Apo AI, apolipoprotein B48 (Apo B48), and fatty acid translocase (CD36) was expressed at a significantly higher level in the EPO group than in the PO group (*p* < 0.05); the free fatty acid receptor 4 (GPR120) (*p* = 0.058) and intestinal fatty acid binding protein (I-FABP) (*p* = 0.064) mRNAs showed a trend toward higher expression in the EPO group than in the PO group. Simultaneously, the relative abundances of the Apo AI and I-FABP proteins were significantly lower in the PO group than in the SBO group (*p* < 0.05). However, no significant difference in the FATP4 and CD36 levels was observed between the SBO and PO groups. Compared with the PO group, the relative abundances of the FATP4 (*p* = 0.031), CD36 (*p* < 0.05), and I-FABP (*p* = 0.065) proteins were higher in the EPO group.

### 3.4. AMPK/mTORC1 and AMPK/Sirt1/NF-κB Pathways in the Jejunum

**Figure 4:**
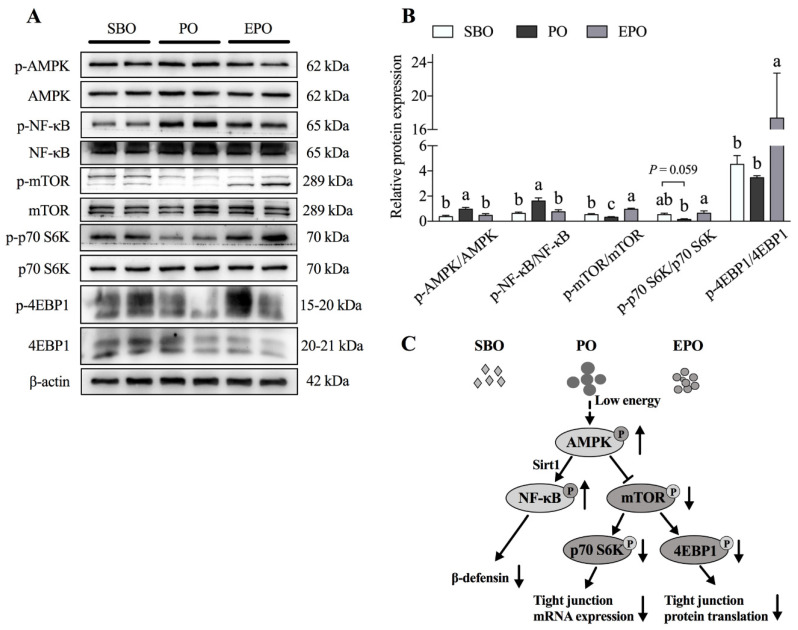
AMPK/mTORC1 and AMPK/Sirt1/NF-κB pathways in the jejunum. (**A**,**B**) The levels of phosphorylated proteins in the AMPK/mTORC1 and the AMPK/Sirt1/NF-κB pathways in the jejunum. (**C**) The energy produced by lipid metabolism regulates the pig intestinal barrier through the AMPK/mTORC1 and AMPK/Sirt1/NF-κB pathways. SBO, soybean oil group; PO, palm oil group; EPO, encapsulated palm oil group. Different letters (a, b, and c) indicate a significant difference among different groups (*p* < 0.05).

We examined the activation of the AMPK/mTORC1 and AMPK/Sirt1/NF-κB pathways in porcine jejunum to elucidate the underlying mechanisms of the effect of the dietary administration of soybean oil, palm oil, and encapsulated palm oil on intestinal function (Figure 4A,B). Our results showed a higher phosphorylation of AMPK and NF-κB in the PO group than in the SBO group (*p* < 0.05). Interestingly, when the animals were treated with encapsulated palm oil, the phosphorylation of AMPK and NF-κB was significantly decreased compared with the PO group (*p* < 0.05). Compared with the SBO group, the phosphorylation of mTOR (*p* < 0.05) and p70 S6K (*p* = 0.059) was lower in the PO group. However, the phosphorylation of mTOR, p70 S6K, and 4EBP1 was significantly increased in the EPO group compared with the PO group (*p* < 0.05).

## 4. Discussion

The intestinal epithelium is a selective permeability barrier that effectively absorbs nutrients and protects the internal environment from the penetration of harmful molecules (such as pathogens, toxins, and antigens) [15]. Intestinal barrier disruption will increase the intestinal cell permeability and the penetration of proinflammatory factors in the intestinal cavity, thereby inducing the activation of the intestinal mucosal immune system and leading to intestinal inflammation and tissue damage [4,16,17]. After weaning, drastic changes in the diet structure and incomplete intestinal development might disrupt the intestinal flora balance, induce intestinal inflammation, and even result in diarrhea in piglets. In addition, early weaning may increase intestinal permeability and reduce the expression of tight junction proteins in piglets [18]. Thus, nutritional strategies designed to enhance the intestinal barrier function are crucial during the weaning period. 

Dietary fat is generally added to diets to provide energy. Fatty acids from dietary fat participate in various metabolic reactions [19,20,21]. However, the effects of fatty acids with different chain lengths differ. Short-chain fatty acids (SCFAs) maintain intestinal biological functions such as energy recovery, cell proliferation, and intestinal barriers [22,23,24,25]. The addition of sodium butyrate to the diet of weaned piglets reduces the incidence of diarrhea because the butyrate treatment increases the mRNA expression of the occludin and ZO-1 in the intestinal mucosa [26]. Similarly, Peng et al. [27] reported that butyric acid increases tight junction integrity and promotes Ca^2+^-induced tight junction assembly in Caco-2 cells. Medium-chain fatty acids (MCFAs) are used directly by intestinal cells to generate energy, which helps support the integrity of piglet intestines [28]. Long-chain fatty acids (LCFAs) ameliorate intestinal inflammation by inhibiting the excessive release of proinflammatory factors [19,20,29], particularly n-3 polyunsaturated fatty acids (PUFAs), arachidonic acid (ARA), and conjugated linoleum acid (CLA) [30,31,32]. The n-3 and n-6 polyunsaturated fatty acids (PUFAs), such as DHA, reduce inflammatory damage to the occludin and ZO-1 [33]. In addition, compared with corn oil, the addition of 5% fish oil to the diet improves the intestinal morphology and increases the expression of the tight junction proteins occludin and claudin-1 [34]. As shown in our previous study, when 6% palm oil is added to the diet, the diarrhea rate of piglets in the palm oil group is significantly higher than that in the soybean oil group (Table S3) [35], which may be related to the substantial difference in fatty acid composition (saturated and unsaturated fatty acids) between soybean oil and palm oil. AMPK is significantly activated in the small intestine of piglets fed palm oil, potentially due to the energy deficiency caused by the low digestibility of PO. AMPK is upstream of mTORC1 and functions as a critical regulator of protein synthesis, including tight junction proteins. In this experiment, claudin expression and mTORC1 signaling pathway activity were inhibited upon the administration of palm oil. Furthermore, the microencapsulation of palm oil activated the mTORC1 signaling pathway and increased the expression of claudin and ZO-1 compared with palm oil in the present study. This evidence suggests that the regulation of intestinal tight junctions by different types of oil may be partially related to their digestibility in the intestine. 

Fatty acids are absorbed by the transportation system in intestinal epithelial cells and then transported into the bloodstream via the lymphatic circulation through the interaction with lipoproteins. Intriguingly, proteins involved in fatty acid absorption and transportation are also regulated by palm oil administration due to energy deficiency. Multiple proteins in the intestinal fatty acid transport system have been identified. For example, FAT/CD36, FATP4, and I-FABP are membrane transporters involved in fatty acid transport. Apo AIV is a glycoprotein that is involved with fat absorption. It is secreted by the intestine and interacts with chylomicron. In this experiment, the microencapsulation of palm oil also increased the expression of CD36, FATP4, I-FABP, and Apo AIV compared with the palm oil. 

In addition to regulating intestinal mucosal permeability and fatty acid transport, fatty acids also affect intestinal defensin secretion [29]. A clear correlation between the fatty acid chain length and the efficiency of induced defensin expression has been identified. When the carbon number of the fatty acid is four or less, the defensin-inducing activity gradually increases as the carbon number increases [36]. However, when the carbon number of a fatty acid exceeds four, its ability to induce defensin activity decreases as the carbon number increases [36]. Caprylic acid and nonanoic acid enhance the immune barrier function of the intestinal epithelium by increasing the expression of the pBD-1 and pBD-2 genes [37]. Octanoic acid and nonanoic acid regulate β-defensin expression by inhibiting the histone deacetylase (HDAC) activity [37]. These results collectively imply that fatty acid-induced β-defensin expression depends on the fatty acid type. Thus, a study of the effects of fat sources and/or types on β-defensin expression is interesting. In the present study, the encapsulation of palm oil significantly increased the duodenal, jejunal, and ileal pBD-2 secretion compared with the palm oil treatment, which may be due to the modification of oil digestion and absorption efficiency in the intestine. Previously, pigs fed encapsulated palm oil were reported to display increased average daily gain (ADG) and G:F levels compared with pigs fed palm oil without encapsulation [35]. In addition, increased fat digestibility is also accompanied by an increased utilization of dry matter (DM) and GE. Thus, fat encapsulation might regulate nutrient digestibility and the activity of AMPK, which modulate the expression of pBD-2 through SIRT1/NF-κB signaling. Consistent with these findings, NF-κB signaling was inhibited by palm oil treatment compared with encapsulated palm oil treatment in this trial. The enhanced physical barrier and immune barrier functions might partially explain how lipid encapsulation alleviates diarrhea occurrence. 

## 5. Conclusions

Fat is an important energy source in animal production. Studies of the effect of oil on gut function will facilitate the development and utilization of new fat sources. In summary, our experiments confirmed that dietary fat regulates the expression of fatty acid transport proteins, intestinal tight junction proteins, and β-defensins. Moreover, we have shown for the first time that fat encapsulation reduces the incidence of diarrhea partially by alleviating the damage to the intestinal mechanical and immunological barriers induced by fat with a low digestibility. At the same time, we hypothesize that the improvement in gut function mediated by microencapsulated fat may be related to the increased efficiency of fatty acid utilization through the AMPK/mTORC1 and AMPK/Sirt1/NF-κB pathways.

## Figures and Tables

**Table 1:** Real-time PCR primer sequence.

Genes	Accession	Direction	Sequences (5′–3′)	Size
Apo AI	NM_214398.1	Forward	CGTGTATGTGGATGCGATCAAA	104
		Reverse	CCCAGTTGTCCAGGAGTTTCAG	
Apo AIV	AJ222966.1	Forward	TCAACACCCAGGTTCAGCAG	121
		Reverse	AACTCATCCGCATAGGGTGC	
Apo B48	AH001207.2	Forward	GAGGTAACTGTGCCTGCATA	436
		Reverse	CTGCACTAAGGTCACGATGT	
GPR120	HQ662564.1	Forward	CTCTTCCTGCTCATGATCTCCTTC	178
		Reverse	GTGACATGTTGTAGAGAATGGGGTT	
FATP4	XM_003353676.1	Forward	AGCCGCATCCTGTCCTTT	213
		Reverse	GACATCCTTGGCGATCTTTT	
CD36	DQ192230.1	Forward	GGACTCAT TGCTGGTGCTGT	169
		Reverse	GTCTGTAAACTTCCGTGCCTGT	
I-FABP	NM_001031780.1	Forward	GAGCTTGGTGTCACTTTTAACTAC	192
		Reverse	CCTCTTGGCTTCTACTCCTTCATA	
IL-FABP	NM_214215.2	Forward	GGAGGTCTCCACTGTCGG	115
		Reverse	CTGTTACACCAGGTTTATTTG	
L-FABP	NM_001004046.2	Forward	GAAGGGGAAGGACATCA	138
		Reverse	CAGTCAGGGTCTCCATCTCACA	
Claudin	NM_001244539.1	Forward	TCTTTCTTATTTCAGGTCTGGCTATCT	247
		Reverse	CCACTGGAAGGCGAAGGTTTT	
Occludin	NM_001163647.2	Forward	ACTGGCGGCGAGTCCTGCGACGAGC	244
		Reverse	TATTGTATTCATCAGCAGCAGCCAT	
ZO-1	XM_021098856.1	Forward	AAAGCCCTAAGTTCAATCACAATCT	253
		Reverse	TCCTCATCTTCATCATCTTCTACAG	
pBD-1	NM_213838.1	Forward	TGCCACAGGTGCCGATCT	81
		Reverse	CTGTTAGCTGCTTAAGGAATAAAGGC	
pBD-2	NM_214442.2	Forward	CCAGAGAGGTCCGACACTACA	168
		Reverse	GGTCCCTTCAATCCTTGTAGGTGAA	
pBD-3	XM_021074698.1	Forward	ACCAAGCACGCCTTCCTATC	236
		Reverse	GCATTTTCGGCCACTCACAG	
pBD-114	NM_001129973.1	Forward	TGTACCTTGGTGGATCCTGAACGA	240
		Reverse	CGCCCTCTGAATGCAGCATATCTT	
pBD-129	NM_001129975.1	Forward	CAAAGACCACTGTGCCGTGAATGA	131
		Reverse	TTGATGCTGGCGAAAGGGTTGGTA	
β-actin	397563	Forward	TGCGGGACATCAAGGAGAAG	176
		Reverse	AGTTGAAGGTGGTCTCGTGG	

Apo AI, apolipoprotein AI; Apo AIV, apolipoprotein AIV; Apo B48, apolipoprotein B48; FATP4, fatty acid transport protein 4; CD36, fatty acid translocase; GPR120, G protein coupled receptors 120; I-FABP, intestinal fatty acid binding protein; IL-FABP, ileum fatty acid binding protein; L-FABP, liver fatty acid binding protein; pBD-1, porcine β-defensin 1; pBD-2, porcine β-defensin 2; pBD-3, porcine β-defensin 3; pBD-114, porcine β-defensin 114; pBD-129, porcine β-defensin 129.

**Table 2:** Antibody information used in this experiment.

Antibodies	Code No.	Company	kDa
Apo AI	ab64308	Abcam	27
Apo AIV	ab239480	Abcam	43
FATP4	ab200353	Abcam	72
CD36	D161529	Sangon	53
I-FABP	ab60272	Abcam	15
Claudin	ab15098	Abcam	23
Occludin	ab31721	Abcam	59
ZO-1	ab96587	Abcam	187
AMPK	2532S	Cell Signaling Technology	62
p-AMPK	2535S	Cell Signaling Technology	62
NF-κB	10745-1-AP	Proteintech Group	65
p-NF-κB	3033S	Cell Signaling Technology	65
mTOR	2983S	Cell Signaling Technology	289
p-mTOR	5536S	Cell Signaling Technology	289
p70 S6K	9202S	Cell Signaling Technology	70, 85
p-p70 S6K	9234S	Cell Signaling Technology	70, 85
4EBP1	ab2606	Abcam	20–21
p-4EBP1	9451S	Cell Signaling Technology	15–20
β-actin	bs-0061R	Bioss	42

## Data Availability

All data used in the current study are available from the corresponding author on reasonable request.

## Supplementary Materials

The following materials are available online at https://www.mdpi.com/2076-2615/11/1/28/s1: Table S1: Composition and nutrient content of experimental diets (as-fed basis) [35], Table S2: Analyzed fatty acid compositions of the fat sources used in the experiment [% (wt/wt) of total fatty acids] [35], and Table S3: Diarrhea incidence in piglets [35].
