# Supplementary material for: Fat Encapsulation Reduces Diarrhea in Piglets Partially by Repairing the Intestinal Barrier and Improving Fatty Acid Transport
Source: Animals (Basel). 2020 Dec 26;11(1):28. doi: 10.3390/ani11010028 (PMC7824132; doi:10.3390/ani11010028)
Supplement: Supplementary file 1 [file animals-11-00028-s001.pdf]

animals

MDPI

Supplementary Materials:

Table S1. Composition and nutrient content of experimental diets (as-fed basis)

Item	Treatments		
	Soybean oil [1]	Palm oil	Encapsulated palm oil [1]
Ingredient, %			
Corn	62.30	62.30	62.30
Soybean meal, 43% CP	4.20	4.20	4.20
Whey powder, 3.8% CP	5.86	5.86	5.00
Fish meal, 62% CP	3.50	3.50	3.50
Soybean oil	6.00	-	-
Palm oil	-	6.00	-
Encapsulated palm oil	-	-	7.50
Fermented soybean meal	12.00	12.00	12.00
Dried casein	0.64	0.64	-
Acidifier	0.60	0.60	0.60
Dicalcium phosphate	0.45	0.45	0.45
Calcium formate	1.34	1.34	1.34
Salt	0.30	0.30	0.30
L-lysine HCl (78.8%)	0.68	0.68	0.68
DL-methionine (99%)	0.37	0.37	0.37
L-threonine (99%)	0.31	0.31	0.31
L-tryptophan (98.5%)	0.08	0.08	0.08
Vitamin premix [2]	0.05	0.05	0.05
Trace element premix [3]	0.10	0.10	0.10
Choline chloride 50%	0.16	0.16	0.16
Chromic oxide [4]	0.35	0.35	0.35
Others [5]	0.71	0.71	0.71
Total	100.00	100.00	100.00
Calculated nutrient composition [6]			
DE, Mcal/kg	3.63	3.59	3.60
CP, %	17.01	17.01	17.01
Ether extract， %	8.43	8.43	8.43
Ash, %	3.52	3.52	2.52
Ca, %	0.70	0.70	0.70
P, %	0.48	0.48	0.48
AP, %	0.33	0.33	0.33
Total lysine, %	1.40	1.40	1.40

SID Lys	1.25	1.25	1.25
SID Met + Cys, %	0.76	0.76	0.76
SID Thr, %	0.81	0.81	0.81
SID Trp, %	0.23	0.23	0.23
Analyzed composition			
GE, Mcal/kg	4.40	4.58	4.50
CP, %	17.05	17.12	17.20
Ether extract, %	8.01	8.68	8.40
Lysine	1.43	1.46	1.47
Methionine + Cystine	0.75	0.77	0.79
Threonine	0.85	0.86	0.88
Tryptophan	0.26	0.24	0.26
Isoleucine	0.70	0.69	0.72
Leucine	1.39	1.37	1.39
Valine	0.87	0.87	0.89
Histidine	0.51	0.45	0.44
Phenylalanine	1.01	0.89	0.92
Arginine	1.23	1.16	1.19

[1] Control diet contains 6.0% soybean oil; encapsulated palm oil and encapsulated coconut oil (containing 80% ether extract) were produced by combing palm oil or coconut oil with dried casein and whey powder in a spray-drying process causing the milk proteins to encapsulate the fat particles as they dried. Dried casein and whey powder were obtained from the same source, other treatment diets without encapsulated fat contained identical amounts of casein and whey.
[2] Provided the following amounts of vitamins per kilogram of diet: vitamin A, 12,000 IU as retinyl acetate; vitamin D_3, 3,600 IU as cholecalciferol; vitamin E, 150 IU as DL-α-tocopherol acetate; vitamin K_3, 7.2 mg as menadione; thiamine, 3 mg; riboflavin, 10.8 mg; pyridoxine, 5.4 mg; vitamin B_{12}, 0.06 mg; pantothenic acid, 36.0 mg; niacin, 60.0 mg; folic acid, 6 mg; biotin, 0.6 mg.
[3] Provided the following amounts of trace minerals per kilogram of diet: Cu, 10 mg as $CuSO_4{\cdot}5H_2O$; Fe, 39 mg as $FeSO_4$. H_2O; Mn, 30 mg as $MnSO_4$; Zn, 39 mg as $ZnSO_4$; Se, 0.15 mg as Na_2SeO_3; I, 0.14 mg as $Ca(IO_3)_2$; Co, 0.1 mg as $CoCl_2$.
[4] The diets of Exp. 2 are the same as in Exp. 1 with additional 3.5kg/T chromic oxide as an inert marker.
[5] Others contains antibiotics, enzymes, mycotoxin removal agent, sweetening agents, zeolite (carrier).
[6] Based on nutrient composition of feed ingredients according to NRC (2012).

Table S2. Analyzed fatty acid composition of the fat sources used in the experiment [% (wt/wt) of total fatty acids][1]

Fatty acid, %	Soybean oil	Palm oil	Encapsulated palm oil [2]
C6:0	0.05	0.09	0.11
C8:0	ND[3]	0.74	0.71
C10:0	0.10	0.74	0.72
C12:0	0.05	5.21	4.66
C14:0	0.11	3.23	3.01
C16:0	11.96	51.23	51.87
C16:1 (cis-9)	0.16	0.15	0.17

C17:0	0.11	0.15	0.14
C18:0	4.02	5.33	5.22
C18:1 (cis-9)	23.83	25.89	26.65
C18:2 (all-cis-9, 12)	52.71	5.83	5.41
C18:3 (all-cis-9, 12,15)	4.81	ND	ND
C20:0	0.42	0.35	0.39
C20:1 (cis-11)	0.28	0.09	0.13
C22:0	0.41	0.49	0.48
Other[4]	0.98	0.48	0.33

[1]Means of two replicates.
[2]The tested fat samples of encapsulated palm oil (80% palm oil) and encapsulated coconut oil (80% coconut oil) were extracted with petroleum ether first at 65 °C for 1 h using an automatic fat extractor and analyzed accordingly.
[3]ND = not detected.
[4]Comprised of 0.5% or less of each of the following fatty acids including: C14:1 (cis-9), C15:0, C17:1 (cis-10), C18:3 (all-cis-6, 9, 12), C24:0.

Table S3. Effect of fat encapsulation on growth performance of nursery pigs

Items	Treatments			SEM
	SBO	PO	EPO	
Body weight, kg				
d 0	8.28	8.10	8.19	0.17
d 28	16.28	15.35	17.09	0.41
ADG, g	268[ab]	241[b]	300[a]	9
ADFI, kg	427	417	437	9
G:F	0.63[ab]	0.57[b]	0.67[a]	0.01
Diarrhea incidence, %	10.68[b]	14.44[a]	7.46[b]	0.93

ADG, average daily gain; ADFI, average daily feed intake; SBO, soybean oil; PO, palm oil; EPO, encapsulated palm oil; SEM, standard error of means.
Different letters (a, b, c) show a significant difference among different treatments ($P < 0.05$).
